# Longitudinal changes in electrical stapedius reflex thresholds (eSRT) in children with cochlear implants

**DOI:** 10.1007/s00405-025-09532-4

**Published:** 2025-07-04

**Authors:** Franz Muigg, Simone Graf, Maria Magdalena Mair, Josef Seebacher, Hazem Salloum, Viktor Weichbold, Joachim Schmutzhard, Philipp Zelger

**Affiliations:** 1 University Hospital for Hearing, Speech & Voice Disorders, Medical University of Innsbruck, Innsbruck, Austria; 2 Department of Otorhinolaryngology-Head and Neck Surgery, Medical University of Innsbruck, 6020 Innsbruck, Austria

**Keywords:** Audiology, Cochlear implant, Electrically evokelectrically evoked stapedius reflex

## Abstract

**Study goal:**

The electrically evoked stapedius reflex threshold (eSRT) is widely used as an estimate of the maximum comfort level (MCL) in the fitting of cochlear implants (CI). This study investigated the long-term evolution of the eSRT during a ten-year observation period.

**Methods:**

Retrospective analysis of fitting map data (charge units [QU] required to evoke the stapedius reflex) obtained from 50 cochlear-implanted ears of 26 children. Time-series statistical methods were employed to examine trends in charge unit (QU) values and their variance, normalized to individual baselines.

**Results:**

The QU time series mean increased significantly within the first six months post-implantation and reached a plateau thereafter. Variance in eSRT decreased significantly over the first five years, with three key turning points identified at six months, 18 months, and 4.25 years, indicating significantly diminishing variation with time.

**Discussion:**

CI fitting schedules should be coordinated with temporal changes in eSRT variance. Frequent fittings in the first 18 months, followed by annual fittings until four years after implantation, seem advisable. After this period, less frequent fittings may be sufficient.

## Introduction

The electrically evoked stapedius reflex threshold (eSRT) is widely used as an objective measure for cochlear implant programming in young children and, generally, in CI-patients who cannot give reliable feedback about their auditory perceptions. It serves as an estimate for the maximum comfortable level (MCL), i.e. the upper limit of electrical stimulation of the acoustic nerve. Stimulation above this level can cause auditory discomfort to the patient.

The suitability of the eSRT as an estimate of the MCL is based on a number of studies that show a high correlation between the two measures, both in children and adults, and regardless of whether the MCL was assessed behaviourally or psychoacoustically (e.g. [1–9]). Yet the suitability of the eSRT as an estimate of the MCL depends also on the temporal evolution of the eSRT. It must be warranted that the eSRT does not undergo large changes over time so that its function as a reliable estimate of the upper stimulation levels in cochlear implants is affected.

Altered eSRT values may lead to worsened CI performance. Studies have shown that a well-fitted CI, including a correctly determined upper limit of the dynamic range, contributes significantly to the audiological outcome of cochlear implantation [10–12]. Wearing a poorly fitted CI for some time bears the risk of getting reduced benefits from the device. For this reason, there is a need to investigate the temporal evolution of the eSRT in order to determine the appropriate time-points of CI fitting.

Several studies have already examined the changes of eSRT over time in pediatric CI samples. However, most of them had a limited observation period, e.g. one year [5, 6, 13–16] or 18 months [17]. Only one study had an observation period of three years [18], but in this study “three years” refers to the period of data collection and not to the period of CI use after implant activation. Therefore, this study also included patients who had received their CI several years before study onset.

In summary, the results of these studies are not fully homogenous. While some of them found, that the eSRT significantly increased during the first three months of CI use and then reached a plateau [14, 17], others reported that only minor (not-significant) changes would occur [5, 6, 18]. Consistent with the former there are reports that electrode impedances are increasing within the first months after CI activation, before remaining on a static level after three to eight months (e.g. [19, 20]).

Based on these (and other) results, it has been recommended that CI adjustments be performed more frequently during the first year of CI use, while thereafter, when the major changes in eSRT have subsided, annual or even less frequent adjustments are sufficient. This recommendation is based on the assumption that after one year no significant eSRT changes occur anymore. This assumption, however, is not sufficiently supported by studies, especially for periods beyond three years of CI use. Against this background, the aim of our study was to investigate the course of the eSRT in pediatric patients during a ten-year observation period after implant activation.

## Methods

### Study design

This study is a retrospective analysis of anonymized patient records. Electrical stimulation data were exported from the clinical database of Maestro software (MED-EL). All available fitting maps of the patients included in the study (as created during their fitting sessions) were detailled and the course of the eSRT values over time was analyzed. Approval for the study from the local Ethics Committee for was received.

### Sample

Inclusion criteria for this study were:

1. Complete fitting data available for at least seven years.
2. Regular, at least eight hours of daily CI use (confirmed by Meastro software data logging).
3. No illness or disorder that may interfere with CI activation or CI use.
4. No re-implantation.

With these criteria, 26 children (19 males, 7 females) were included in the study. All but two of them were bilaterally implanted, yielding a total of 50 ears to be analyzed. All children had received a MED-EL CI with one of the following implant types: Synchrony (37), Sonata (10), and Pulsar (3).

**Fig. 1:**
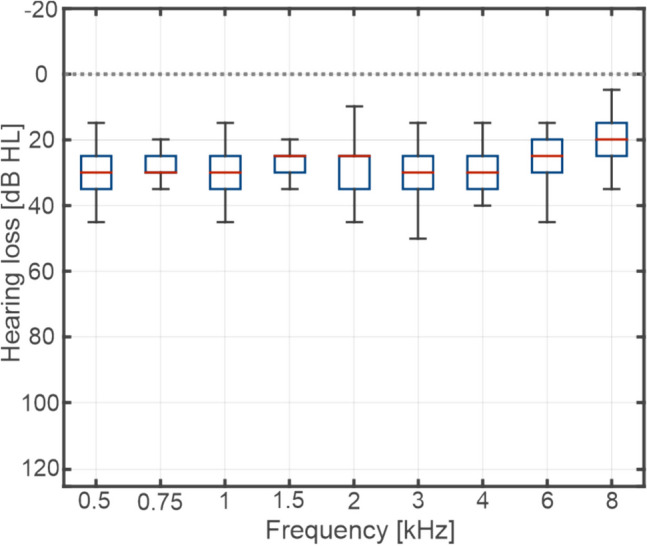
Mean aided hearing thresholds of the sample, measured at the last point in time of the observation period

The sample’s mean age at implantation in the first ear was 2.0 years (range: 0.9 to 3.8 years) and in the second ear 3.4 years (range 1.9 to 5.9). Except for one child who had single-sided deafness, all other children had bilateral sensorineural hearing loss. The hearing loss was assumed to be genetic in all children, as no other causes could be identified. The mean hearing threshold of the sample (pure tone average at frequencies 0.5/1/2/4 kHz, measured at the last point in time of the observation period) was 30 (± 11) dB HL. Figure 1 provided a detailled overwiew of the mean aided hearing thresholds at particular frequencies. The observation period started with CI activation and lasted either 7 years (3 ears), eight years (3 ears), 9 years (2 ears), or 10 years (42 ears).

### Statistics

Since CI fitting was not performed at uniform points in time in all children, a time series statistics was preferred to a repeated measures design.

The outcome measure was the Charge Units (QU) required to evoke the stapedius reflex. The QU values were normalized inter-individually by expressing all measurements from a person as relative values of their last measured mean. The last measured mean was designated as “0 relative QU”. From the relative QU values, the trends of the time series mean and of the variance were calculated.

To detect significant changes in the time series mean, the cumulative sum (CUSUM) test was used. The CUSUM test detects shifts or changes in the mean value of a time series.

In order to determine significant changes in the variance of the relative QU values ​​over time, the time series was divided into ten one-year periods and the variance between adjacent periods was compared statistically. Before the statistical comparison, it was checked whether the relative QU values ​​within the period are normally distributed (Kolmogoroff-Smirnov test). Since the normal distribution could not be confirmed in most cases, the (parameter-free) Levene test was used to determine significant differences between the variance of two periods.

To identify the points in time, where the trend in variance significantly changes, the Iterative Cumulative Sum of Squares (ICCS) test was used [21]. The ICCS test detects structural breakpoints in the variance of time series data.

## Results

**Fig. 2:**
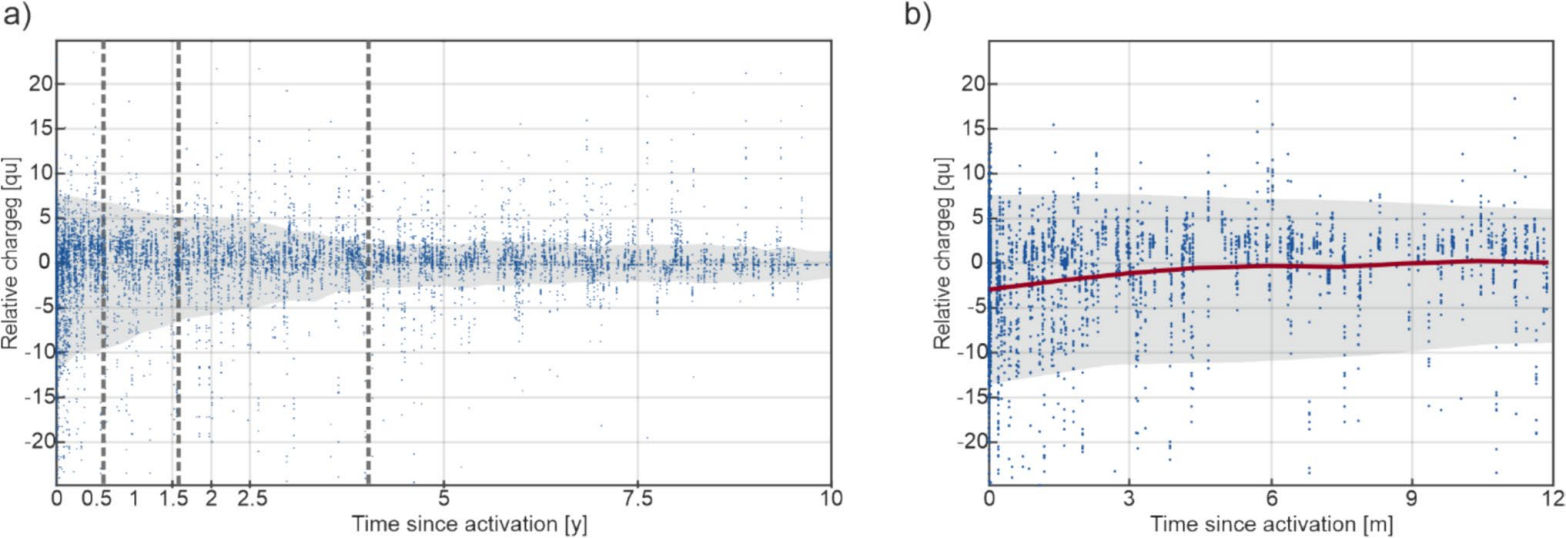
(**a**) Scatter plot of all relative QU values (black points) during a ten-year observation period. The gray zone represents the standard deviations. Dashed vertical lines represent turning points of the temporal trend of the variance. (**b**) Scatter plot of the relative QU values in the first 12 months. The red line shows the relative QU mean during this period

The results are presented graphically in Fig. 2a (ten-year observation period) and Fig. 2b (one-year time window).

Figure 2a shows the distribution of the relative QU values (from all electrode channels of all CIs) during the entire ten-year observation period. The gray area in the figure represents the standard deviation of the time series mean (calculated using a moving average with a window width of 50 days). Visual inspection of Fig. 2a shows clearly that the variance, as indicated by standard deviations, is successively decreasing during the first four years. Afterwards, only little changes are seen. Figure 2b provides a closer look at the data from the first year. It shows that the relative QU mean (red line) increases steadily until about the fourth month. From then on it remained close to the normal (zero-)line.

Statistical comparison of the mean values ​​of the QU time series shows no significant change over the 10-year period (CUSUM test, *p* = 0.36). However, when comparing the changes within the one-year period, a significant increase in the relative QU mean is shown within the first six months (*p* = 0.03).

Similarly, the variance of the relative QU values is found to ​​change significantly over time. The statistical comparison of neighbored one-year periods with the Levene Test indicates that up to five years after implant activation, every annual decrease is significant (with all p’s < 0.005) while thereafter no more significant changes are seen.

When analyzing the temporal course of the variance with the ICSS test, statistically significant turning points in the temporal trend are found at six months, 18 months, and 51 months (marked as vertical dashed lines in Fig. 2a). At these points in time, the decline in the variance slows down significantly until it no longer changes after 51 months.

## Discussion

This study used time series statistics to analyze the course of the eSRT during a period of ten years in cochlear-implanted children. Its goal was to detect significant temporal changes in eSRT and eSRT variances in order to gain insights that are helpful in the scheduling CI fitting appointments.

As for the first year of CI use, the findings of our study confirm the findings of previous studies that the eSRT is, on average, decreased after CI surgery and increases significantly in the first few months. While other studies have reported that the increase continues up to three months [17] or even up to eight months [19] after CI activation, our study found that eSRT levels reach a plateau after six months.

However, it is to note that eSRT values ​​show considerable variation over time, both between channels and between individuals. For this reason, also their variance (as a measure of variation) has to be considered. Decreases in variance are additional indicators that eSRT is a robust estimator of upper stimulation levels, hence our study also looked for significant changes in the variance. It was found, on the one hand, that the variance steadily decreased in the first five years of CI use, with significant annual differences. After that, it remained more or less on a static level. On the other hand, three turning points of variance change were identified: after six months, after 18 months and after 4.25 years. At these time points, there was a significant reduction in eSRT variability, suggesting that CI fitting may be required less frequently from this point onwards due to the higher eSRT stability.

Beyond five years, no significant changes were observed in either the mean or variance of the relative QU values across the time series. In summary, our study suggests the following fitting schedule: At least three fitting sessions should occur within the first six months after implant activation, followed by sessions every three months until 18 months post-activation. Between 18 months and about four years, annual fittings appear sufficient, although pediatric patients may require more frequent adjustments due to other factors. After four years of CI treatment, even fewer fitting intervals, eg. bi- or three-annually, may suffice.

This recommendation holds significant clinical relevance, as it can contribute to more efficient use of resources for both clinics and families. Fitting sessions are time-consuming and often require patients, particularly children and their parents, to travel long distances to specialized centers. Reducing the number of these sessions to only those that are necessary can help ease the burden on families while allowing clinical staff to allocate their time more effectively.

However, to safely minimize fittings, clinicians must have reliable information on how frequently adjustments are truly needed. Until now, such recommendations have often been based on general clinical assumptions rather than longitudinal data. By analyzing the long-term development of eSRT thresholds, our study provides empirical evidence that may help guide the scheduling of fitting sessions more precisely.

At the same time, our findings emphasize the importance of maintaining some degree of objective monitoring. While mean eSRT thresholds appear stable at the group level, individual patients may still experience fluctuations due to developmental, biological, or medical factors. Continued monitoring of eSRTs helps ensure that important changes in stimulation needs are not overlooked—especially in populations where behavioral assessments can be limited by age, language barriers, or cognitive comorbidities.

eSRT-based fitting provides a stable objective measure that reflects physiological responsiveness to electrical stimulation compared to subjective fitting methods (e.g. the subjective loudness perception of patients). Changes in subjective perception (e.g., due to temporary factors like fatigue or headaches) may not reflect true changes in auditory function, whereas shifts in eSRTs could point to underlying clinical or device-related issues that warrant further investigation.

Therefore, our study not only offers a basis for optimizing fitting schedules but also reinforces the role of eSRTs as a clinically meaningful tool in individualized cochlear implant management.

Pioneered by Stephan [22] and by Jerger [23], the eSRT has become an important method for the estimation of upper stimulation levels in CI patients who are not able to provide reliable feedback on their auditory perceptions. The importance of the eSRT may even increase in the future, as studies indicate that alternative methods for estimation of upper stimulation levels, such as electrically evoked compound action potential (ECAP) or loudness scaling, are inferior to the eSRT. ECAPs correlate less well with the upper loudness limit and are therefore considered less reliable estimates of the MCL than eSRT [9, 24, 25]. Loudness scaling, on the other hand, can only be used in cooperative patients. It may intuitively seem that loudness scaling is more reliable in determining the upper loudness limit than eSRT, but a recent study suggests that eSRT-based fitting maps provide better audiological outcomes than maps based on loudness scaling [26]. If this finding is confirmed by further studies, then estimation of MCL through eSRT could become the generally preferred method even in patients with good introspection skills.
